# Dental health and lung cancer risk in the Golestan Cohort Study

**DOI:** 10.1186/s12885-024-11850-5

**Published:** 2024-01-13

**Authors:** Yukiko Yano, Christian C. Abnet, Gholamreza Roshandel, Akua Graf, Hossein Poustchi, Masoud Khoshnia, Akram Pourshams, Farin Kamangar, Paolo Boffetta, Paul Brennan, Sanford M. Dawsey, Emily Vogtmann, Reza Malekzadeh, Arash Etemadi

**Affiliations:** 1 Division of Cancer Epidemiology and Genetics, National Cancer Institute, National Institutes of Health, Bethesda, MD USA; 2 Golestan Research Center of Gastroenterology and Hepatology, Golestan University of Medical Sciences, Gorgan, Iran; 3 Liver and Pancreatobiliary Diseases Research Center, Digestive Diseases Research Institute, Tehran University of Medical Sciences, Tehran, Iran; 4 Digestive Oncology Research Center, Digestive Diseases Research Institute, Tehran University of Medical Sciences, Tehran, Iran; 5 Department of Biology, School of Computer, Mathematical, and Natural Sciences, Morgan State University, Baltimore, MD USA; 6 Stony Brook Cancer Center, Stony Brook University, Stony Brook, NY USA; 7 Department of Medical and Surgical Sciences, University of Bologna, Bologna, Italy; 8 Section of Genetics, International Agency for Research on Cancer, World Health Organization, Lyon, France

**Keywords:** Oral health, Tooth loss, Tooth decay, Toothbrushing, Lung cancer

## Abstract

**Background:**

Poor oral health has been linked to various systemic diseases, including multiple cancer types, but studies of its association with lung cancer have been inconclusive.

**Methods:**

We examined the relationship between dental status and lung cancer incidence and mortality in the Golestan Cohort Study, a large, prospective cohort of 50,045 adults in northeastern Iran. Cox proportional hazards models were used to estimate hazard ratios (HRs) and 95% confidence intervals (CIs) for associations between three dental health measures (i.e., number of missing teeth; the sum of decayed, missing, or filled teeth (DMFT); and toothbrushing frequency) and lung cancer incidence or mortality with adjustment for multiple potential confounders, including cigarette smoking and opium use. We created tertiles of the number of lost teeth/DMFT score in excess of the loess adjusted, age- and sex-specific predicted numbers, with subjects with the expected number of lost teeth/DMFT or fewer as the reference group.

**Results:**

During a median follow-up of 14 years, there were 119 incident lung cancer cases and 98 lung cancer deaths. Higher DMFT scores were associated with a progressively increased risk of lung cancer (linear trend, *p* = 0.011). Compared with individuals with the expected DMFT score or less, the HRs were 1.27 (95% CI: 0.73, 2.22), 2.15 (95% CI: 1.34, 3.43), and 1.52 (95% CI: 0.81, 2.84) for the first to the third tertiles of DMFT, respectively. The highest tertile of tooth loss also had an increased risk of lung cancer, with a HR of 1.68 (95% CI: 1.04, 2.70) compared with subjects with the expected number of lost teeth or fewer (linear trend, *p* = 0.043). The results were similar for lung cancer mortality and did not change substantially when the analysis was restricted to never users of cigarettes or opium. We found no associations between toothbrushing frequency and lung cancer incidence or mortality.

**Conclusion:**

Poor dental health indicated by tooth loss or DMFT, but not lack of toothbrushing, was associated with increased lung cancer incidence and mortality in this rural Middle Eastern population.

**Supplementary Information:**

The online version contains supplementary material available at 10.1186/s12885-024-11850-5.

## Background

Poor oral health is a major global public health problem [1]. Around 3.5 billion people worldwide are affected by oral diseases, predominantly untreated dental caries (tooth decay), severe periodontal disease, and tooth loss [2]. These oral conditions not only impact the health of the teeth and mouth but also systemic health [3]. Periodontal disease has been associated with various systemic diseases, such as diabetes, cardiovascular disease, and cancer [3, 4]. Observational studies have repeatedly shown associations between tooth loss– often resulting from periodontal disease– and several cancer types, particularly cancers of the upper gastrointestinal tract [5, 6]. Regular oral hygiene practices, namely toothbrushing, have been associated with a decreased risk of developing certain cancers [7, 8]. These associations between poor oral health and systemic diseases, including cancer, are suspected to share a common pathway mediated by the oral microbiome [9]. The mechanism of these associations may involve carcinogenic bacterial metabolites (e.g., acetaldehyde produced by ethanol-metabolizing oral microbes [10], and nitrosamines formed from nitrate reduced to nitrite by nitrate-reducing oral microbes [11, 12]), chronic systemic inflammation triggered by the oral microbiome, or specific periodontal pathogens and their interplay with the host immune response [9].

Several prospective studies have previously reported adverse associations between poor oral health, as measured by tooth loss and/or periodontal disease, and lung cancer incidence or mortality [13–15]. However, the relationship between oral health and lung cancer risk remains inconclusive, particularly since smoking may modify associations. Some studies found that smokers may have greater risk of lung cancer if they have poor oral health [14, 16, 17], and other studies found no significant associations between oral health and lung cancer in never smokers [14, 17–21]. In addition, many of the existing studies have been from the United States [17, 20–22] where smoking is a common exposure that may have altered the relationship between oral health and lung cancer. The current evidence is lacking studies from diverse populations, particularly studies from prospective cohorts outside of the US with adjustment for smoking and other major confounders of lung cancer associations.

Here, we examined the association between poor dental health and lung cancer incidence and mortality in the Golestan Cohort Study, a large-scale, population-based prospective study with more than 50,000 participants in Golestan Province, located in northeastern Iran. We used multiple dental health measures, including tooth loss; the sum of decayed, missing, or filled teeth (DMFT score); and frequency of toothbrushing, to investigate the impact of poor dental health on lung cancer risk.

## Methods

### Study population and questionnaire data

As described in detail previously [23], the Golestan Cohort Study is a prospective, population-based cohort of 50,045 individuals between ages 40 and 75 years at baseline in Golestan Province, Iran. Participants were recruited from January 2004 to June 2008 and continue to be followed up. Written informed consent was obtained from all study participants at the time of enrollment. The Golestan Cohort Study was approved by the Institutional Review Boards of the Digestive Disease Research Institute of Tehran University of Medical Sciences, the International Agency for Research on Cancer, and the United States National Cancer Institute.

At baseline, participants were interviewed in-person by trained staff using a structured questionnaire to collect sociodemographic and lifestyle information, including age, sex, ethnicity, place of residence, education, and detailed information on the use of cigarettes, nass (a local chewing tobacco product), and opium (e.g., age at initiation and cessation and amount of use per day). Opium consumption is a known carcinogen [24] and risk factor for different cancers including lung cancer [25]. Individuals who use opium are exposed to most of the carcinogens present in tobacco smoke [26]. Fruit and vegetable intake were assessed at baseline using a food frequency questionnaire. Socioeconomic status (SES) was estimated based on a composite wealth score determined by ownership of vehicles, property, and household appliances [27]. The high reliability and validity of self-reported cigarette smoking and opium use in this population have been demonstrated previously [28, 29].

### Dental health assessment

As part of the baseline interview, trained medical staff counted each participant’s total number of teeth and the number of decayed, missing, or filled teeth, the sum of which constitutes the DMFT score. Participants were also asked about toothbrushing habits, and toothbrushing frequency was categorized as never, non-daily, and daily. The reliability of tooth counts and self-reported brushing frequency have both been shown to be high in this population [8, 30]. Specifically, a pilot study was previously conducted for the Golestan Cohort Study where the reliability of teeth counts was evaluated based on repeated examinations of 130 participants occurring two months apart [30]. These results showed that the reliability of the teeth counts was high, with 88.3% agreement and a kappa statistic of 0.86. Similarly, the reliability of self-reported toothbrushing frequency has been evaluated based on a subset of the cohort (11,418 randomly selected participants) who completed a repeat questionnaire approximately 5 years after the baseline interview where participants were asked how often they brush their teeth [8]. The self-reported toothbrushing frequency at baseline and from the repeated assessment showed excellent agreement with 77.9% concordance (*p* < 0.001). The maximum number of teeth and DMFT score were coded as 32 to represent the total number of adult teeth including third molars because these are not routinely extracted in this population.

### Case ascertainment

All study participants were followed annually through telephone surveys or home visits, and provincial death and cancer registry data were reviewed monthly to identify all incident cancers and deaths due to any cause. In the case of death, a validated verbal autopsy was performed where the closest relative of the deceased was interviewed by a trained physician to obtain information about the cause of death [31]. Cancer diagnoses and deaths were confirmed by linking to the Golestan population-based cancer registry [32]. Primary lung cancer was defined using International Classification of Diseases, Tenth Revision (ICD-10) codes C34.0-C34.9. Six subjects diagnosed with nonepithelial malignancies (i.e., 4 subjects with lymphoma and 2 subjects with neuroendocrine carcinoma) of the lung were excluded from the present analysis.

### Statistical analysis

Of the 50,045 cohort participants, 9 subjects missing dental status variables and 83 subjects with other missing covariates were excluded, in addition to the 6 subjects with nonepithelial lung cancer diagnoses, leaving a total of 49,947 subjects remaining in the analysis. We used age-dependent exposure metrics to account for the strong correlation between oral variables and age and sex [33]. Specifically, a loess model was fit to estimate the predicted number of lost teeth or DMFT score at each integer year of age, stratified by sex. The loess smoothing parameter was selected based on the bias-corrected Akaike information criterion. Excess numbers of lost teeth and DMFT score were calculated for each participant by taking the difference between the loess predicted age- and sex-specific number of lost teeth/DMFT score and the observed number of lost teeth/DMFT score. Those with a difference of 0 or fewer than the expected number were categorized into the reference group, and the remaining subjects with excess tooth loss/DMFT were categorized into tertiles.

Cox proportional hazards regression models were used to estimate hazard ratios (HRs) and 95% CIs for the association between oral health variables (i.e., tooth loss, DMFT, and toothbrushing frequency), other potential risk factors (described below) and lung cancer incidence and mortality. The entry time was defined as the date of enrollment into the Golestan Cohort Study. Follow-up ended on the date of lung cancer or other cancer diagnosis (for lung cancer incidence analysis only), death, or last follow-up through March 31, 2021, whichever came first. A total of 518 participants (1.04%) were lost to follow-up during the study period.

Cox models were run separately for each dental health variable, including the following sociodemographic and lifestyle variables: age, sex, SES (in quartiles) [27], ethnicity (Turkmen or non-Turkmen), residence (urban or rural), education (illiterate or literate), nass use (never or ever), cigarette use, and opium use. For cigarette smoking, participants were categorized as never smokers or in tertile categories of their cumulative pack-years of smoked cigarettes, with separate analyses run for former and current smokers. Cumulative pack-years of cigarette smoking was calculated as the number of packs (20 cigarettes in each pack) smoked per day multiplied by the number of years of smoking. For opium use, participants were categorized as never users or in tertile categories of their number of years of consumption. We further performed analyses stratified by cigarette smoking and opium use (never smoker/opium user or ever smoker/opium user) and tested for interactions between oral health variables and smoking/opium use (coded as a binary variable of never or ever smoker/opium user) using the likelihood ratio test. Dental health variables were tested for a linear trend by assigning ordinal numbers to each category, and the Wald test was used for testing for a global trend. Deviations from the proportional hazard assumption were not detected in any of the models based on the Schoenfeld residuals test. All statistical tests were two-sided with a significance level of 0.05. The R programming environment [34] (version 4.2.2) was used for all statistical analyses.

## Results

Table 1 shows the baseline characteristics of the cohort participants, overall and by DMFT category. The majority of cohort participants had never smoked cigarettes (82.8%) or used nass (92.3%) or opium (83.1%). Overall, the mean cigarette smoking pack-years was 16.9 (SD 18.6) for ever smokers (mean smoking pack-years was 16.3 [SD 21.0] and 17.3 [SD 16.9] for former and current smokers, respectively), and the mean duration of opium use was 12.2 (SD 10.7) years for ever opium users. The mean number of missing teeth and the mean DMFT score were 18.3 (SD 9.55) and 23.4 (SD 8.73), respectively, and more than half of the cohort participants (55.7%) reported never brushing their teeth. Relative to subjects with the expected DMFT score or lower, a larger proportion of individuals in the highest tertile of DMFT were male, lived in rural areas, smoked cigarettes, and used opium or nass (Table 1). During a median 14 years of follow-up there were 119 incident lung cancer cases (crude incidence rate of 17.9 cases per 100,000 person-years), and 98 of these people died of lung cancer. Of the 119 lung cancer cases, 53 (44.5%) were never cigarette smokers, 66 (55.5%) were never opium users, and 45 (37.8%) used neither.

**Table 1:** Baseline characteristics of the Golestan Cohort Study participants, overall and by DMFT group

			DMFT group			
		Overall	Expected or fewer	T1	T2	T3
N		49,947	22,257 (44.6)	10,082 (20.2)	10,906 (21.8)	6702 (13.4)
Age, years, mean (SD)		51.6 (8.92)	50.1 (8.31)	57.5 (10.7)	52.9 (6.75)	45.3 (3.87)
Sex, n (%)	Female	28,792 (57.6)	12,384 (55.6)	6599 (65.5)	6772 (62.1)	3037 (45.3)
	Male	21,155 (42.4)	9873 (44.4)	3483 (34.5)	4134 (37.9)	3665 (54.7)
SES, quartile, n (%)	Q1 (low SES)	13,909 (27.8)	5655 (25.4)	3116 (30.9)	3186 (29.2)	1952 (29.1)
	Q2	11,125 (22.3)	4611 (20.7)	2304 (22.9)	2535 (23.2)	1675 (25.0)
	Q3	12,567 (25.2)	5465 (24.6)	2514 (24.9)	2871 (26.3)	1717 (25.6)
	Q4 (high SES)	12,346 (24.7)	6526 (29.3)	2148 (21.3)	2314 (21.2)	1358 (20.3)
Ethnicity, n (%)	Turkmen	37,176 (74.4)	16,040 (72.1)	7525 (74.6)	8409 (77.1)	5202 (77.6)
	Non-Turkmen	12,771 (25.6)	6217 (27.9)	2557 (25.4)	2497 (22.9)	1500 (22.4)
Residence, n (%)	Urban	10,006 (20.0)	5496 (24.7)	2057 (20.4)	1744 (16.0)	709 (10.6)
	Rural	39,941 (80.0)	16,761 (75.3)	8025 (79.6)	9162 (84.0)	5993 (89.4)
Education, n (%)	None	35,059 (70.2)	14,122 (63.4)	8135 (80.7)	8665 (79.5)	4137 (61.7)
	Any	14,888 (29.8)	8135 (36.6)	1947 (19.3)	2241 (20.5)	2565 (38.3)
Cigarette use status, n (%)	Never	41,366 (82.8)	18,968 (85.2)	8564 (84.9)	8971 (82.3)	4863 (72.6)
	Former	3193 (6.39)	1271 (5.71)	651 (6.46)	749 (6.87)	522 (7.79)
	Current	5388 (10.8)	2018 (9.07)	867 (8.60)	1186 (10.9)	1317 (19.7)
Cigarette smoking pack-years (former/current smokers only), mean (SD)		16.9 (18.6)	13.8 (16.0)	19.0 (22.5)	21.0 (21.4)	16.5 (14.7)
Opium use, n (%)	Never	41,501 (83.1)	19,267 (86.6)	8333 (82.7)	8838 (81.0)	5063 (75.5)
	Ever	8446 (16.9)	2990 (13.4)	1749 (17.3)	2068 (19.0)	1639 (24.5)
Opium use years (ever users only), mean (SD)		12.2 (10.7)	10.9 (9.85)	13.7 (12.5)	13.5 (11.4)	11.3 (8.71)
Cigarette or opium use, n (%)	Never used either	37,358 (74.8)	17,439 (78.4)	7627 (75.6)	7992 (73.3)	4300 (64.2)
	Ever used either	12,589 (25.2)	4818 (21.6)	2455 (24.4)	2914 (26.7)	2402 (35.8)
Nass use, n (%)	Never	46,077 (92.3)	20,757 (93.3)	9277 (92.0)	10,000 (91.7)	6043 (90.2)
	Ever	3870 (7.75)	1500 (6.74)	805 (7.98)	906 (8.31)	659 (9.83)
DMFT score, mean (SD)		23.4 (8.73)	15.3 (6.20)	28.2 (3.95)	30.7 (2.29)	31.6 (1.08)
Number of teeth missing, mean (SD)		18.3 (9.55)	11.4 (6.11)	23.3 (7.67)	24.8 (7.86)	22.9 (9.02)
Tooth loss group, n (%)	Expected or fewer	26,303 (52.7)	19,466 (87.5)	2783 (27.6)	2463 (22.6)	1591 (23.7)
	T1	8478 (17.0)	2705 (12.2)	3232 (32.1)	1667 (15.3)	874 (13.0)
	T2	7337 (14.7)	86 (0.39)	3978 (39.5)	2509 (23.0)	764 (11.4)
	T3	7829 (15.7)	0 (0)	89 (0.88)	4267 (39.1)	3473 (51.8)
Toothbrushing frequency, n (%)	Never	27,815 (55.7)	10,062 (45.2)	6742 (66.9)	7024 (64.4)	3987 (59.5)
	Non-daily	8341 (16.7)	4756 (21.4)	1296 (12.9)	1320 (12.1)	969 (14.5)
	Daily	13,791 (27.6)	7439 (33.4)	2044 (20.3)	2562 (23.5)	1746 (26.1)

DMFT, the sum of decayed, missing, or filled teeth; Q, quartile; SD, standard deviation; SES, socioeconomic status; T, tertile

**Fig. 1:**
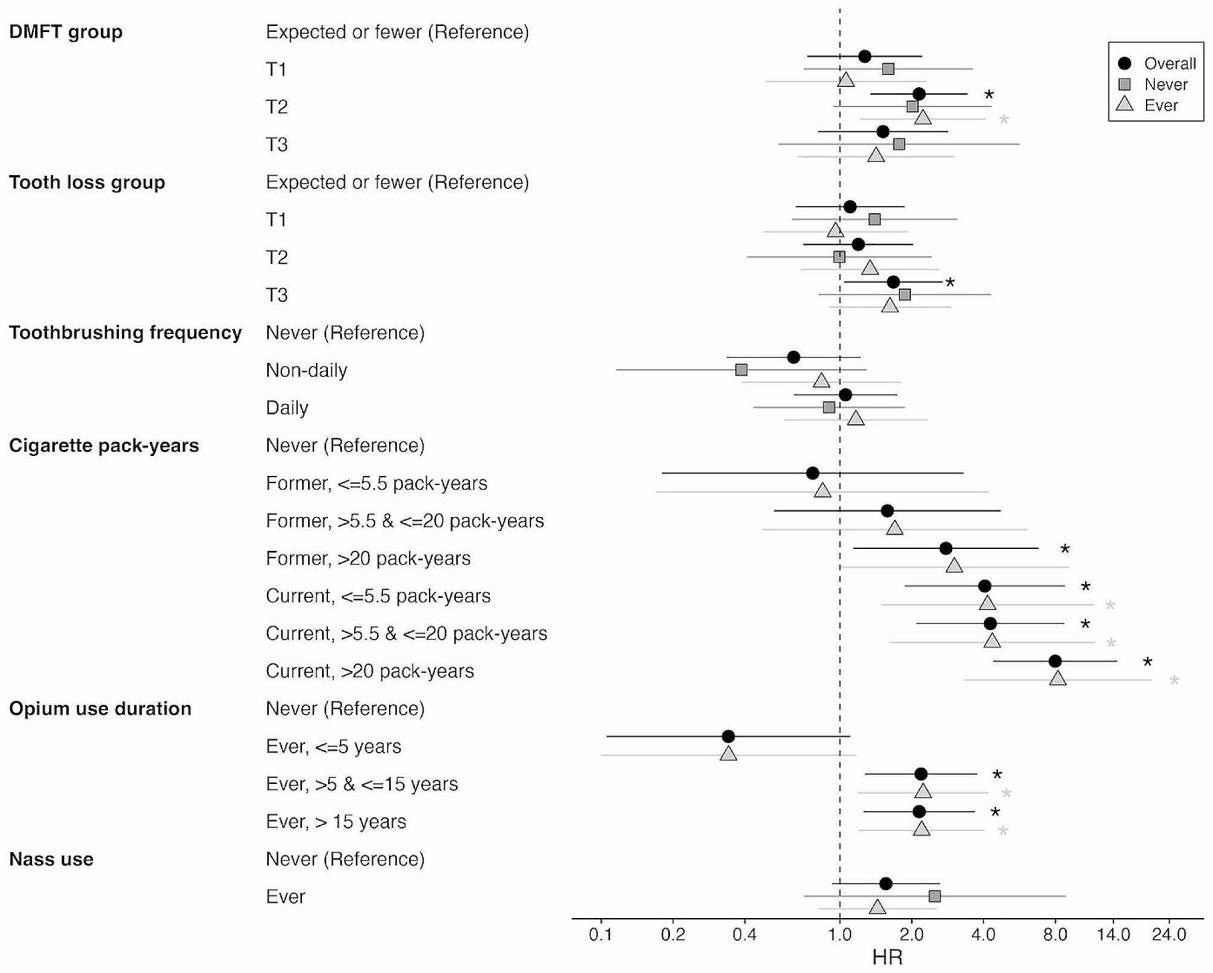
Associations between dental status, substance use, and incident lung cancer, overall and stratified by cigarette smoking and opium use status. Subjects were stratified into binary groups of those that ever smoked cigarettes or used opium and those that never used either cigarettes or opium. DMFT, the sum of decayed, missing, or filled teeth; HR, hazard ratio; T, tertile. HRs for cigarette, opium, and nass use are from the model including DMFT. In the stratified analysis of ever users of cigarettes/opium, the reference group for cigarette pack-years included subjects that never smoked cigarettes but used opium, and vice versa for the opium use reference group. Full results, including associations with sociodemographic factors, can be found in Tables S1 and S2. *P < 0.05

We first examined associations between cigarette smoking, opium use, and nass use and lung cancer incidence (Fig. 1, Table S1). Age, cigarette smoking, and opium use were significantly associated with an increased risk of lung cancer, whereas sex, SES, ethnicity, area of residence, education, and nass use did not have a significant association with lung cancer risk, with mutual adjustment for all potential risk factors including the dental health variables. Compared with never smokers, former smokers with over 20 pack-years of smoked cigarettes had a higher risk of lung cancer (HR 2.78 [95% CI: 1.14, 6.80] in a model that included DMFT), but former smokers with 20 pack-years or less did not. All current smokers had higher lung cancer risk compared with never smokers regardless of the number of pack-years. Current smokers with 5.5 pack-years or less and current smokers with 5.5–20 pack-years had HRs of 4.05 (95% CI: 1.87, 8.75) and 4.27 (95% CI: 2.09, 8.71), respectively. Lung cancer risk was further increased for current smokers with over 20 pack-years with HR of 7.98 (95% CI: 4.39, 14.5). Ever using opium for over 5 years was also significantly associated with an increased lung cancer risk with a HR of around 2.2 compared with never users. Ever use of nass was not significantly associated with an increased risk of lung cancer compared with never use.

Poor dental status was associated with an increased risk of incident lung cancer (Fig. 1, Table S1) in models adjusted for known and suspected lung cancer risk factors. Specifically, there was an increasing trend in lung cancer risk across the DMFT tertiles (linear trend, *p* = 0.011; global trend, *p* = 0.011). Relative to individuals with the expected DMFT score or less, the HR increased from 1.27 (95% CI: 0.73, 2.22) to 2.15 (95% CI: 1.34, 3.43) across the first two tertiles of DMFT but dropped to 1.52 (95% CI: 0.81, 2.84) for the highest tertile (Fig. 1, Table S1). The highest tertile of tooth loss was also associated with an increased lung cancer risk with a HR of 1.68 (95% CI: 1.04, 2.70) compared with subjects with the expected number of lost teeth or fewer, but no associations were found for the first two tertiles of tooth loss (linear trend, *p* = 0.043; global trend, *p* = 0.19) (Fig. 1, Table S1). There were no significant associations between toothbrushing frequency and lung cancer risk (Fig. 1, Table S1).

We further examined associations between dental status, other potential risk factors, and lung cancer incidence, stratified by cigarette smoking and opium use, important risk factors in this population. Subjects were stratified into binary groups of never (*n* = 37,358; 45 cases) and ever (*n* = 12,589; 74 cases) users of cigarettes or opium. For the non-oral health related risk factors (i.e., age, sex, SES, ethnicity, area of residence, education, former and current smoking pack-years, and opium and nass use), the results did not change upon stratification (Table S1). For DMFT, the results were similar among never and ever cigarette/opium users, with significant associations for the second tertile of DMFT (Fig. 1, Table S2). For never smoker/opium users, HRs were 1.59 (95% CI: 0.71, 3.60), 2.02 (95% CI: 0.94, 4.33), and 1.77 (95% CI: 0.55, 5.66) from the first to the third tertile of DMFT. For ever smoker/opium users, HRs were 1.06 (95% CI: 0.49, 2.30), 2.23 (95% CI: 1.21, 4.09), and 1.42 (95% CI: 0.66, 3.03) from the first to the third tertile of DMFT. Strata-specific HRs were similar to the overall unstratified HRs (2.15 for the second DMFT tertile; Fig. 1, Table S1). Stratification also did not change the results for tooth loss or toothbrushing frequency (Fig. 1, Table S2). We found no evidence of a statistical interaction between smoking/opium use and any of the dental status variables (*p* > 0.49).

For lung cancer mortality, associations with dental health variables were similar to those for incidence but had slightly elevated risk estimates for DMFT (Table S3, Fig. S1). The second tertile of DMFT was significantly associated with an increased risk of lung cancer mortality, with a HR of 2.55 (95% CI: 1.50, 4.33), and mortality risk significantly increased with higher DMFT tertiles (linear trend, *p* = 0.0038; global trend, *p* = 0.0046). For tooth loss, the highest tertile of tooth loss had a HR of 1.71 (95% CI: 1.01, 2.92), and there was a marginally significant linear trend across the tertiles of tooth loss (*p* = 0.049). Associations with toothbrushing frequency remained null for lung cancer mortality.

Sensitivity analyses excluding the first two years of follow-up did not meaningfully change the Cox regression analysis results for either lung cancer incidence or mortality (Table S4, Fig. S2). Excluding subjects with no teeth (8,709 subjects with no teeth, including 34 incident lung cancer cases) did not change the results for associations between DMFT and lung cancer incidence, but associations with tooth loss and toothbrushing frequency were null (Table S5, Fig. S3). Adjusting for daily fruit and vegetable intake also did not substantially change associations with lung cancer incidence (Table S6, Fig. S3).

## Discussion

In this large, prospective cohort study, more than half of the cohort members reported never brushing their teeth, and the participants had on average 23.4 decayed, missing, or filled teeth. Higher DMFT scores were associated with a progressively higher risk of both lung cancer incidence and mortality, and the second tertile of individuals with higher-than-expected DMFT score had more than a two-fold risk of lung cancer compared with subjects who had the expected DMFT score or less. Similarly, there was a ~ 1.7-fold increased risk of lung cancer for subjects in the highest tertile of increased tooth loss compared with those with the expected number of lost teeth or fewer. These dental health variables were significantly associated with lung cancer risk after simultaneous adjustment for other risk factors, including age, cigarette smoking, and opium use. We found no associations between toothbrushing frequency and lung cancer risk.

Our results from the Golestan Cohort Study show that poor dentition (i.e. higher numbers of tooth loss or higher DMFT score) is independently associated with lung cancer risk, and it is unlikely that these results can be explained by residual confounding by tobacco or opium use. This is in line with previous studies of tooth loss and lung cancer, with a recent meta-analysis including seven studies showing a relative risk of 1.64 (95% CI: 1.44, 1.86) comparing the highest and lowest category of tooth loss for incident lung cancer [14]. Tooth loss often results from periodontal disease, which has also been shown to be associated with an increased risk of lung cancer in multiple prospective cohort studies (meta-analyzed HR of 1.40 (95% CI: 1.25, 1.58) [35]). Also similar to our results, a cohort study in Japan found that higher numbers of teeth lost were associated with an increased risk of lung cancer mortality (0–9 teeth remaining vs. 20 or more teeth remaining, HR 1.75; 95% CI: 1.08, 2.83), with adjustment for smoking and other potential confounders [36]. However, there have also been other studies, such as the prospective cohort analysis of the Sister Study cohort in the US, that did not find a significant association of periodontal disease or tooth loss with lung cancer mortality [37].

Almost half of the lung cancer cases in our study were never smokers. In addition, more than half of the cases had never used opium, which is another known lung cancer risk factor that is relevant in this population [25], and 37.8% used neither cigarettes nor opium. We furthermore showed that associations with dental status remained largely unchanged upon stratification by smoking status and opium use. In previous cohort studies, some found no significant associations between poor oral health (tooth loss and/or periodontal disease) and lung cancer incidence [14, 17, 19, 20] or mortality [21] in never smokers but found poor oral health to increase risk for current [14] or former [17] smokers. It is possible that smoking may modify associations between poor oral health and lung cancer risk, but more studies are needed to clarify this.

The mechanism for the association between oral health and lung cancer likely involves the oral microbiome. Oral microbes produce various metabolites that have been linked to carcinogenesis, such as acetaldehyde [10], nitrosamines [38], and reactive oxygen species [9]. Some authors have suggested that edentulism and the healing of gum tissue may ameliorate the negative effects of tooth loss by altering the oral microbiome against the overgrowth of bacterial species that produce carcinogenic metabolites [39], but we did not find strong evidence to support this hypothesis when we excluded subjects with no teeth from the analysis (Table S5). The oral microbiome can also impact cancer risk at distant sites through systemic inflammation, which is a key component of both periodontal disease and carcinogenesis [40, 41]. Recently, there have been a few studies that have found potential links between the oral microbiome and lung cancer. A case-cohort study of three US cohorts found that greater diversity in the oral microbiome was associated with lower risk of developing lung cancer, and relative abundances/presence of certain genera were associated with risk; for example, higher relative abundances of *Streptococcus* was associated with increased lung cancer risk [42]. In addition, two nested case-control studies (one from a low-income population in the southeastern US [43] and another among never smokers in China [44]) found different specific taxa to be associated with increased or decreased lung cancer risk. Another recent nested case-control study conducted in the US found that serum antibodies to 13 periodontal bacteria were mostly inversely associated with lung cancer risk, possibly indicating immunity against certain bacteria that may help reduce cancer risk [45]. Additional types of evidence beyond observational studies are warranted to understand the exact mechanism of association between poor oral health, the oral microbiome, and lung cancer.

Our study has several strengths and limitations. The major strengths of this study include its prospective design and low loss to follow-up. We used multiple measures to evaluate dental status, which were assessed by trained interviewers. However, our study did not examine the participants’ periodontal status, so we could not evaluate the effect of this component of poor oral health. We carefully adjusted (and stratified by when necessary) for multiple potential confounders, including cigarette smoking, opium use, and SES, but, as with all observational epidemiologic studies, our findings may have been impacted by unmeasured confounders or residual confounding. We also accrued a limited number of lung cancer cases and this precluded analysis by histology and restricted power. Finally, all dental health measures were ascertained at a single time point and accounting for changes in dental status over the follow-up period might have led to different exposure ranking of cohort members.

## Conclusion

We found evidence in this cohort that poor dental status, as indicated by higher DMFT scores and greater tooth loss, was associated with an increased risk of lung cancer incidence and mortality after controlling for other important risk factors such as cigarette smoking and opium use. These results persisted even when the analysis was restricted to never users of cigarettes or opium. We did not find significant associations for toothbrushing frequency. While known risk factors such as smoking and opium use remain important, our results indicate that poor oral health may also contribute to lung cancer risk.

### Electronic supplementary material

Below is the link to the electronic supplementary material.

**Supplementary Material 1:** Supplementary tables

**Supplementary Material 2:** Supplementary figures

## Data Availability

The data that support the findings in this study are available from the corresponding authors upon request.
