# Supplementary material for: Dental health and lung cancer risk in the Golestan Cohort Study
Source: BMC Cancer. 2024 Jan 13;24:74. doi: 10.1186/s12885-024-11850-5 (PMC10787979; doi:10.1186/s12885-024-11850-5)
Supplement: Supplementary file 2 — Supplementary Material 2: Supplementary figures [file 12885_2024_11850_MOESM2_ESM.docx]

**Supplemental material**

**Dental health and lung cancer risk in the Golestan Cohort Study**

Yukiko Yano^1^, Christian C. Abnet^1^, Gholamreza Roshandel^2^, Akua Graf^1^, Hossein Poustchi^3,4^, Masoud Khoshnia^2^, Akram Pourshams^3,4^, Farin Kamangar^5^, Paolo Boffetta^6,7^, Paul Brennan^8^, Sanford M. Dawsey^1^, Emily Vogtmann^1^, Reza Malekzadeh^3,4^, Arash Etemadi^1,4^

1. Division of Cancer Epidemiology and Genetics, National Cancer Institute, National Institutes of Health, Bethesda, Maryland, USA
2. Golestan Research Center of Gastroenterology and Hepatology, Golestan University of Medical Sciences, Gorgan, Iran
3. ﻿Liver and Pancreatobiliary Diseases Research Center, Digestive Diseases Research Institute, Tehran University of Medical Sciences, Tehran, Iran
4. Digestive Oncology Research Center, Digestive Diseases Research Institute, Tehran University of Medical Sciences, Tehran, Iran
5. ﻿ Department of Biology, School of Computer, Mathematical, and Natural Sciences, Morgan State University, Baltimore, Maryland, USA
6. Stony Brook Cancer Center, Stony Brook University, Stony Brook, NY, USA
7. Department of Medical and Surgical Sciences, University of Bologna, Bologna, Italy
8. Section of Genetics, International Agency for Research on Cancer, World Health Organization, Lyon, France

**Figure S1.** Associations of dental status and substance use with lung cancer mortality.

**
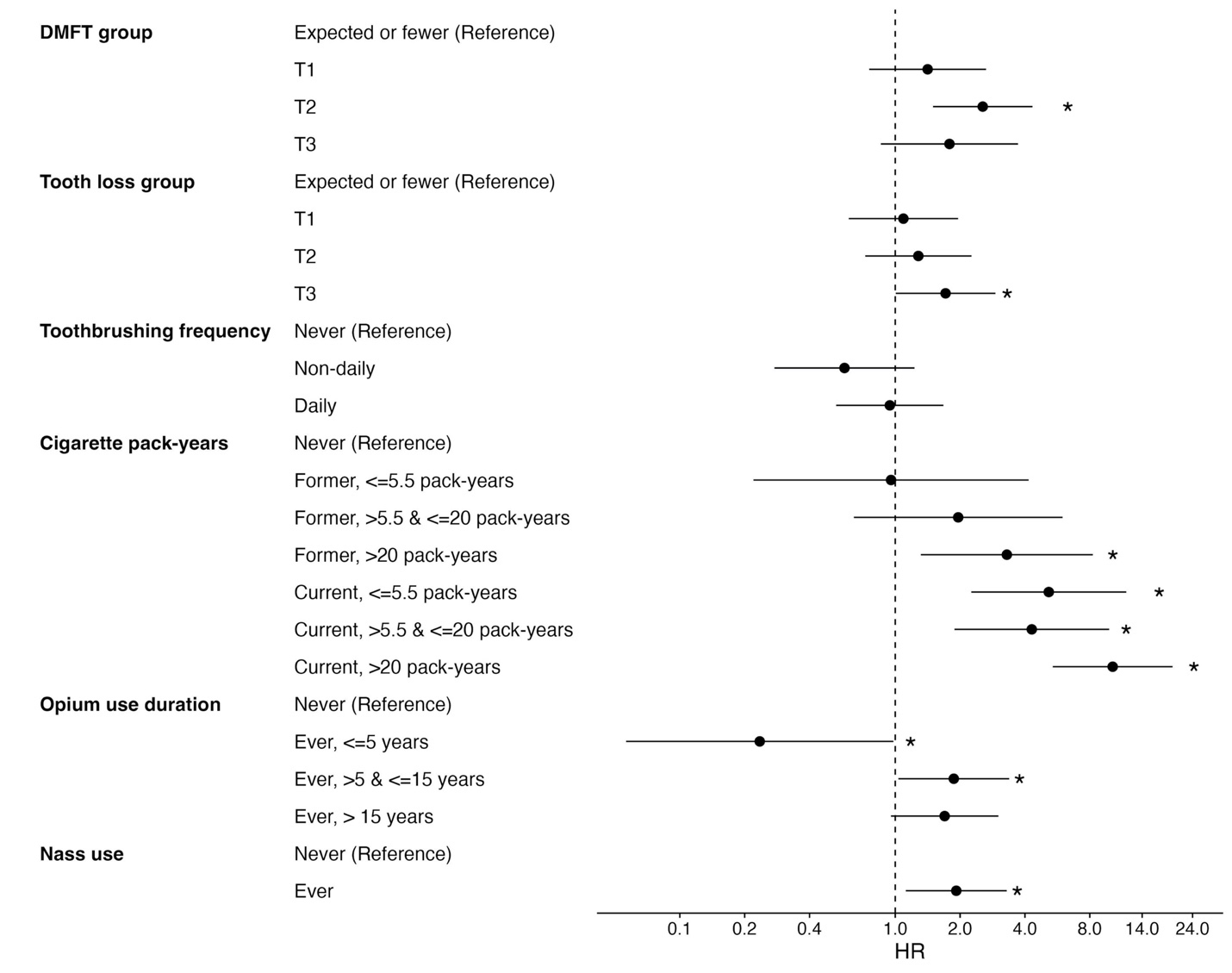
**

DMFT, the sum of decayed, missing, or filled teeth; HR, hazard ratio; T, tertile.

HRs for cigarette, opium, and nass use are from the model including DMFT.

Full results, including associations with sociodemographic factors, can be found in Table S3.

* P < 0.05

**Figure S2.** Associations of dental status and substance use with lung cancer incidence and mortality, excluding the first two years of follow-up.


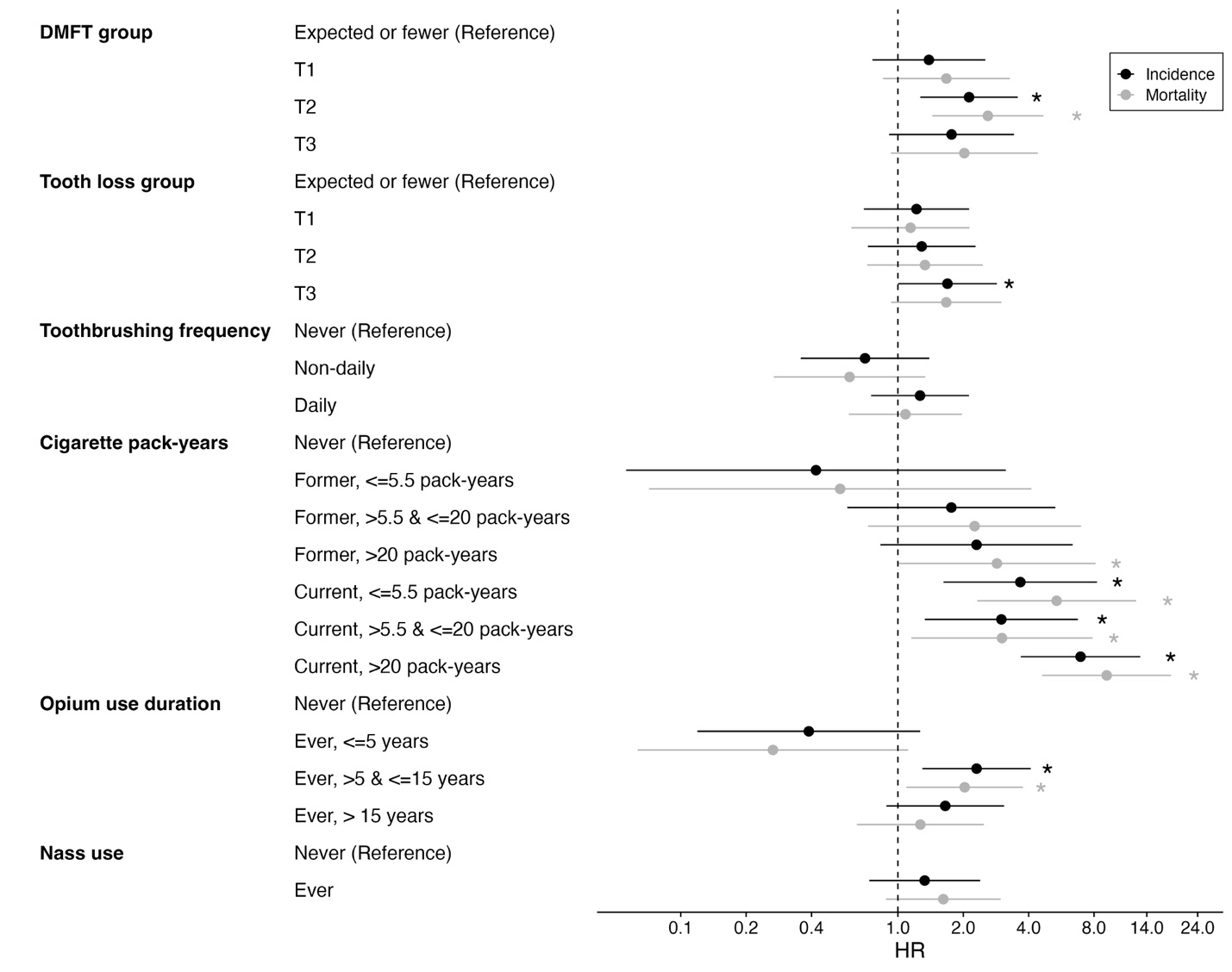


DMFT, the sum of decayed, missing, or filled teeth; HR, hazard ratio; T, tertile.

HRs for cigarette, opium, and nass use are from the model including DMFT.

Full results, including associations with sociodemographic factors, can be found in Table S4.

* P < 0.05

**Figure S3.** Associations of dental status and substance use with lung cancer incidence excluding subjects with no teeth (edentulism) and lung cancer incidence adjusted for daily fruit and vegetable intake.


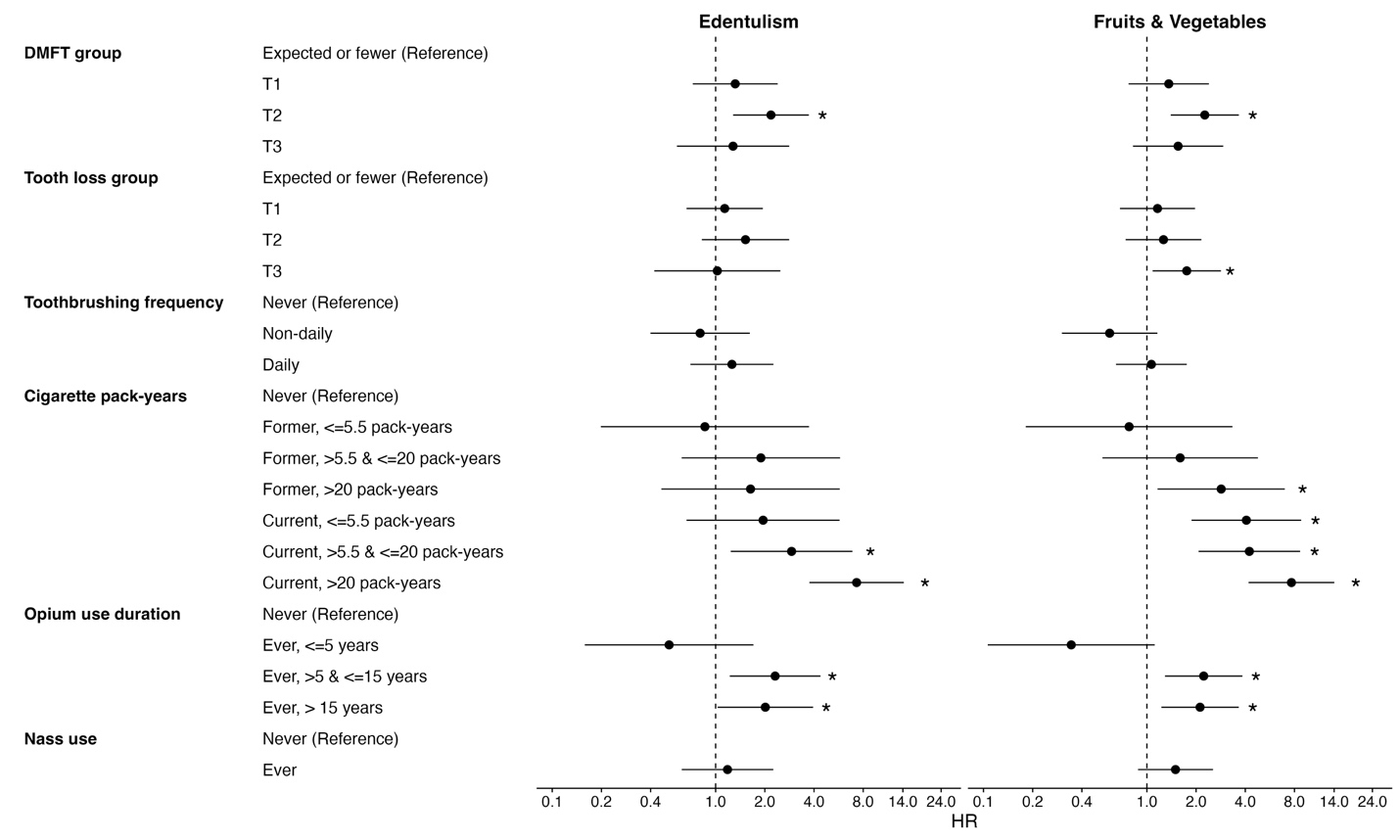


DMFT, the sum of decayed, missing, or filled teeth; HR, hazard ratio; T, tertile.

HRs for cigarette, opium, and nass use are from the model including DMFT.

Full results, including associations with sociodemographic factors, can be found in Table S5 and S6.

* P < 0.05
